# Official Report of the Transactions of the American Medical Association at Its Recent Meeting in Pamphlet Form

**Published:** 1870-06

**Authors:** 


### Official Report of the Transactions of the American Medical Association at its Recent Meeting in Pamphlet Form.

The peımanent Secretary of the American Medical Association, Dr. William B. Atkinson, has issued an official report of the proceedings of the recent meeting in Washington. There was a great necessity for this, as nothing appeared except the newspaper reports, which were incorrect. The real action of the association has not yet been published, and we are satisfied that the profession will feel under the greatest obligation to Dr. Atkinson for thus early furnishing the report in pamphlet form. It can be obtained by addressing him at No. 1400 Pine street, Philadelphia, Pa. *Price*, 25 *cents.*

We hope that all our readers will avail themselves of this report, and if they have time, compare it with our report of the "legislative section," *mostly from notes taken on the spot*, though the Secretary, in a private note intimates that "*all* the reports thus far published, are based upon newspaper reports, which are very erroneous." The permanent Secretary has done more for the association than any other man; contributed most of all to its interest, prosperity, and usefulness, and we hope to never say less of him, whatever he may think of the accuracy of our observations at the last association meeting, but would very modestly suggest that *we* published our report from original personal observation, and though the association voted against any papers being carried into or out of the hall, thus confiscating our notes, still the report was *original.* Hope the Secretary will make the acknowledgment, and that all physicians will send for his report.

